# Metallic Magnetic Nanoparticles

**DOI:** 10.1100/tsw.2005.121

**Published:** 2005-12-22

**Authors:** A. Hernando, P. Crespo, M. A. García

**Affiliations:** Instituto de Magnetismo Aplicado, Depto. de Física de Materiales, Universidad Complutense, P.O. Box 155, Las Rozas, Madrid, Spain

**Keywords:** metallic nanoparticles, nanomagnetism, size effects

## Abstract

In this paper, we reviewed some relevant aspects of the magnetic properties of metallic nanoparticles with small size (below 4 nm), covering the size effects in nanoparticles of magnetic materials, as well as the appearance of magnetism at the nanoscale in materials that are nonferromagnetic in bulk. These results are distributed along the text that has been organized around three important items: fundamental magnetic properties, different fabrication procedures, and characterization techniques. A general introduction and some experimental results recently obtained in Pd and Au nanoparticles have also been included. Finally, the more promising applications of magnetic nanoparticles in biomedicine are indicated. Special care was taken to complete the literature available on the subject.

### GENERAL REMARKS

#### Introduction

As has been recently pointed out, the new measuring techniques — as well as the refinement in synthesis and preparation methods allowing the fabrication of nanometric particles — are in the basis of the explosion of this interdisciplinary branch known as nanoscience[1]. However, the study of the physical properties of "small" systems has been of interest to many scientists for a long time. The term "small" should be understood as small in dimensions relative to the typical lengths of some physical phenomenon. For instance, as concerns magnetism, the term "small particle" can account for particles with dimensions smaller than the domain wall thickness, but also can account for particles with volume small enough to enhance the importance of surface energy terms (wall energy) respective to those proportional to the volume (magnetostatic energy), giving rise to a single domain structure. For both cases, the dimensions are measured in length units that represent typical magnetic lengths. The study of this type of small particles was driven in the 1950s by the interest in finding magnets with ideal hardness, i.e., samples without domain walls that are responsible for a significant softness[2].

In this review, we focus on magnetic properties of extremely small-sized metallic particles, i.e., smaller than 4 nm. Reviews on nanocrystalline materials as well as on magnetic nanoparticles (NPs) inserted in a different kind of matrix are available in the recent literature[3,4]. In particular, magnetic properties of NPs capped with oxide layers have been thoroughly studied and reported[4,5,6]. Along with this introductory section, the general concepts underlying the experimental results recently found in magnetic NPs and described below are discussed.

### Electronic Structure and Size

A more general scaling of metal particle dimensions can be defined from the discreteness of the electronic energy spectrum. As is known, the spacing between adjacent conduction energy states increases inversely with the volume of the particle. Therefore, the continuum that describes the band of allowed energy states becomes a discrete set of states for NPs, as Fig. 1 illustrates. The spacing between adjacent states becomes an energy unit inverse to the cubic root of the particle dimensions. For particles 100 Å in size, the energy-level spacing corresponds typically to 1 K[1]. The discreteness of the spectrum leads to profound modifications in those physical properties depending on the solid electrons, as optical, conduction, and magnetic properties. In particular, the density of states at the Fermi level, the parameter that governs these types of properties, is strongly affected by the nanoscale.

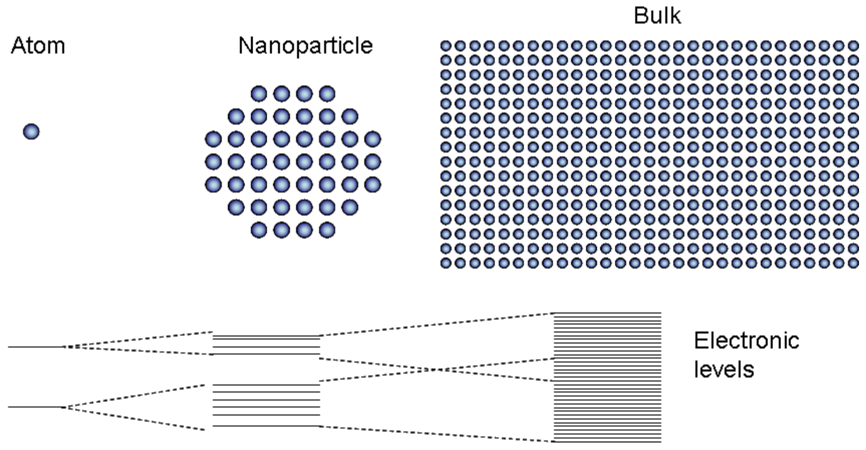

**FIGURE 1.** Evolution of electronic levels from a single atom to the bulk state.

### Influence of Surface on the Physical Properties of Nanoparticles

Another important characteristic of NPs is the enormous surface-to-volume number of atoms. There are two important consequences related to this ratio. For an ideal solid, the surface can be thought of as a defect. The breaking of translational symmetry gives rise to different local physical properties. The electronic states change to surface states, the magnetic anisotropy is modified to surface anisotropy, and the crystalline or amorphous short-range order undergoes a sharp discontinuity as the surface is approached from the internal volume. Thereby, all the physical properties sensitive to short-range order exhibit surface behavior different to that observed in the volume. The solid properties can be roughly envisaged as an average of the surface and volume properties, the relative weights being the surface and volume atoms number fraction. The number fraction of surface atoms, $n_s$, for a solid with dimension L and average interatomic distance $a$ is given by $\alpha a/L$, where $\alpha$ is a sample geometry-dependent parameter ($\alpha = 6$ for a cubic shape). The number fraction of volume atoms, $n_v$, is then given by $1-\alpha a/L$. For bulk samples, $n_v$ is much grater than $n_s$ and the macroscopic properties are those corresponding to volume atoms slightly modulated by surface contribution. For instance, for a cube-shaped particle with a = 1.5 Å and L = 1 mm, $n_s$ is about $10^{-6}$. However, for L = 1.5 nm, $n_s$ is about 0.9 and the average sample properties are expected to be those corresponding to the surface (see Fig. 2). As a consequence of these considerations about the overwhelming influence of surface on physics of NPs, it is interesting to remember that high-resolution electron diffraction microscopy has shown that metallic NPs often exhibit different crystalline symmetry than bulk samples of the same metal. These observations provide direct evidence that the leader role of surface in NPs is strong enough to modify even the crystalline structure. For instance, fcc bulk Pd evolves toward several different structures — as fcc cube-octahedron, icosahedron[7]— when dimensions fall into the nanoscale realm. It is the high surface energy of <100> facets that forces the crystalline order of NPs, breaking the typical bulk symmetry to avoid these characteristic fcc high-energy facets.

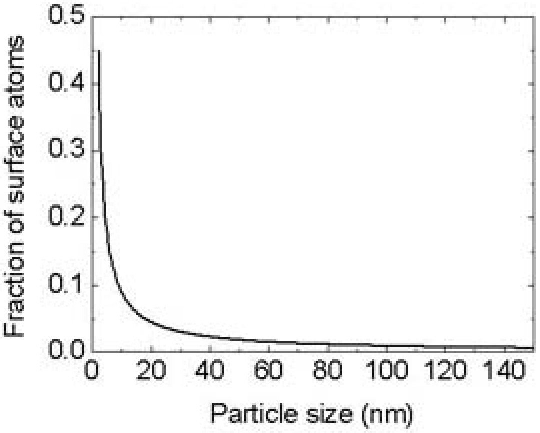

**FIGURE 2.** Fraction of surface atoms as a function of the particle size for cubic shape ($\alpha = 6$) and in teratomic distance a = 1.5 Å.

The second consequence of the governing role of surface is the enormous influence that atoms at the environment exert on the NPs physical properties. It is well known that surface acts as a boundary connecting solids with the external world. In the case of NPs, the solid is formed almost solely by the surface itself. Therefore, the sample as a whole is dramatically sensitive to the atoms coating the particle in several possible different forms. This influence has been experimentally found to be as important as that previously discussed. In bulk solids, the dependence of physical properties on the chemical environment can be defined as weak when compared to that exerted on NPs. Recent experimental studies have shown that physical properties of NPs can be tuned by suitable coating. It seems to be obvious that covalent bond, for instance, of surface atoms of NPs is going to affect drastically their electronic and, consequently, the optical and magnetic properties. In other words, the electronic structure that is extremely sensitive to the nanoscale will also be deeply modified by the chemical bonding of the atoms. Note that coating of NPs by covalent bonding to other types of atoms gives rise to a singular type of chemical compound very different to those we are familiar with in bulk scale. For instance, a Au NP coated with thiol bonding is not expected to behave as Au sulfide, even though a large number of covalent Au-S bonds could be identical for both types of samples[8].

Finally, it is to be understood that NP systems always present size and shape fluctuations. The corresponding distributions are extremely important to understand and model the physical properties[1].

## FUNDAMENTAL MAGNETIC PROPERTIES AFFECTED BY NANOSCALE

### Permanent Magnetism

Let us consider first the magnetic moment of metallic NPs. Permanent magnetic moments would be induced in samples as a consequence of exchange interactions or anisotropy effects. The magnetic moment of a single NP is that obtained by adding the individual magnetic moment of the total number of electrons in the sample. If this resultant moment is different to zero and remains unchanged during the measuring time, it is known as permanent magnetic moment. Ferromagnetic exchange interactions can promote the appearance of spontaneous magnetic moment in the scale of magnetic domains, when the density of states at the Fermi level is sufficiently high. As the electronic energy structure is drastically affected at nanometric sizes, it is possible to observe permanent magnetism in NPs with compositions that are not magnetic in the bulk configuration. This is the case of Pd NPs as described below[9].

More interesting is the possibility of anisotropy-induced, permanent magnetic moment in NPs. Magnetic anisotropy is a local effect at atomic scale. Anisotropy strength increases with spin-orbit coupling and with lack of spherical symmetry in the atomic environment. When the strength of the local anisotropy energy is for a single atom higher than the thermal energy $K_BT$, the magnetic moment is frozen in direction. Even if the orientation of the easy axis was randomly distributed in the NP, a permanent magnetic moment could be measured as a consequence of the small number of atomic moments to be averaged. To show that, let us call *n* the number of atoms at the NP carrying permanent magnetic moment, with strength $\mu$ close to the Bohr magneton, $\mu_B$. The estimation of the average moment for the NP, for a random orientation of the local easy axis, can be inferred from elementary random walk considerations to be $<\mu> = \mu/(n)^{1/2}$. As *n* is a small number for NP with size small, i.e., *n* = 140 for L = 1 nm, $<\mu>$ can reach well-detectable values without requiring the existence of ferromagnetic exchange coupling between the magnetic moments, as Fig. 3 illustrates. This effect is discussed below concerning the surprising permanent magnetism detected in thiol-capped Au NPs[10].

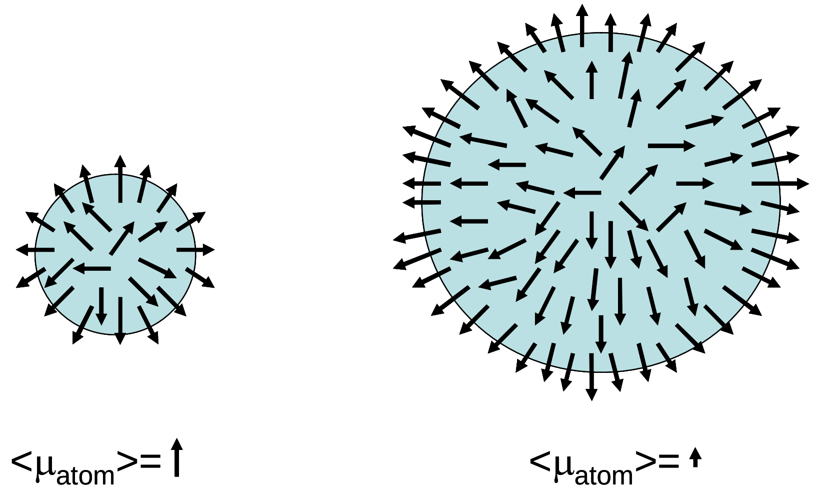

**FIGURE 3.** Picture of the magnetic moment distribution and average magnetic moment per atom for NP of different size.

Some organic monolayers made from diamagnetic molecules adsorbed on diamagnetic substrates exhibit giant magnetization and hysteresis loops like that typical of ferromagnets. These surprising magnetic properties are due to the details of the transference of electrons from the substrate to the organized organic molecules and only can be observed at nanoscale[11,12].

### Ferromagnetic Excitations

As is well known, the first excited states above the ferromagnetic ground state can be analyzed within the framework of the spin wave theory.

It is obvious that, according to the Planck distribution function that governs the number of magnons or elementary excitations, those with larger wavelength are the only ones excited at low temperatures. However, magnons with wavelengths larger than the NP size cannot be excited. Therefore, for any NP size, there exists a temperature threshold below which no spin wave can be induced. For this T range, the spontaneous magnetization cannot decrease by spin wave excitations and the theory would predict a plateau for the thermal dependence of magnetization. Confinement of spin waves giving rise to standing modes have been observed by inelastic neutron scattering in manganites with nanosized ferromagnetic clusters[13]. The influence of shape effect at nanoscale on thermal excitation of magnons has also been studied from both theoretical and experimental viewpoints for ribbon-shaped Fe NPs[14].

Some quantitative considerations would help to understand size and shape influence on spin wave excitations. For a ribbon-shaped NP with length $l$ and square cross-section with side $w < l$, the wave vector, $\boldsymbol{k}$, of magnons with wavelength $\lambda$, comprised between $l$ and $w$, are constrained to lie along the longitudinal direction. However, wave vector of those magnons with $\lambda > w$ becomes 3D vector similar to the bulk. The total number of magnons at temperature T is given by the sum of 1D and 3D total number of allowed $\boldsymbol{k}$ vectors. For 1D, the following number of magnons is easily found[14]:

$$\sum_k n_{1,k} = \frac{1}{2\pi l w^2} \cdot \frac{dk}{e^{\left(\frac{Dk^2}{k_B T} - 1\right)}} \quad (1)$$

whereas for 3D magnons we find:

$$\sum_k n_{3,k} = \frac{hw^2}{2\pi^2 l w^2} \cdot \frac{k^2 dk}{e^{\left(\frac{Dk^2}{k_B T} - 1\right)}} \quad (2)$$

where D is the exchange stiffness constant that, for instance, for bcc Fe, is 0.281 eVA$^2$.

Note that in bulk materials, only the last integral with limits 0 and infinity holds. It has been shown that the total number of magnons can be written as:

$$\sum_k n_{1,k} + \sum_k n_{3,k} = A\left((h-1)\frac{T}{w}\right) - C\left(\frac{T^{5/4}}{w^{1/2}}\right) + B\left(T^{3/2}\right) \quad (3)$$

where $h = l/w$ and A, C, and B are constants that depend on D. The third term describing the total number of excited magnons corresponds to the well-known Bloch law. The first term or A term (that vanishes for h = 1, i.e., for isotropic-shaped NPs ) and the second term or C term, decreases and vanishes as the particle size increases up to infinity. The first term contributes to the enhancement of the number of magnons and describes the shape effect through its dependence on $h$. Fig. 4 shows the thermal dependence of magnetization obtained by using expression (3) for NPs with $w$ = 4 nm and different aspect ratios. The thermal dependence for h = 1 and for bulk sample are also included[15].

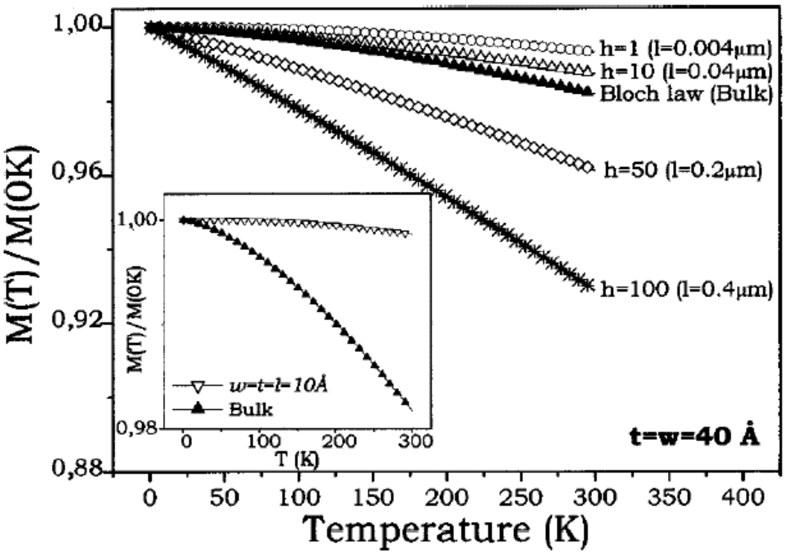

**FIGURE 4**. Thermal dependence of magnetization given by expression (3) for a sample with $t = w = 40$ Å and different aspect ratios, $h$. The inset shows the temperature dependence of the magnetization in the low $T$ and/or $t$ limit for $h = 1$. The thermal dependence for a bulk sample, i.e., Bloch law, is also included. From ref. [14].

In summary, a new framework describing the first excited states of the ferromagnetic ground state should be addressed when the NP size is larger than the magnon wavelength.

### Domains and Domains Walls

Magnetic domains decrease the magnetostatic free energy associated with a particle uniformly magnetized along the easy magnetization direction. This energy term can be expressed as $(1/2)\mu_0 N M_s^2 V$, where N is the demagnetizing factor along the easy axis or spontaneous magnetization, $M_s$, is direction and V is the particle volume. If we consider a domain wall separating two domains with opposite directions along the same easy axis, the corresponding magnetostatic energy is reduced to roughly one-half. However, the domain wall encloses two energy term contributions as due to exchange and anisotropy. Let γ be the sum of these two terms per unit area of the wall, then the total wall energy is given by γS, where S is the total wall area. The increment of energy, Δ, due to domain formation is given by:

$$\Delta = \gamma \cdot S - \frac{1}{4}\mu_0 N M_S^2 V \quad (4)$$

Therefore, domain formation would be energetically favorable for negative Δ. For $(S/V) > \mu_0 N M_s^2 / 4\gamma$, the formation of domains is unfavorable and the particle remains as a single domain. Consequently for any material there exists a limiting size:

$$L_D = \frac{4\gamma}{\mu_0 N M_S^2} \quad (5)$$

For $L < L_D$, the particle is a single domain. $L_D$ is a typical magnetic length to be scaled with NP size.

Let us consider a spherical particle with N = 1/3, $\mu_0 M_s$ = 1T, and $\gamma = (AK)^{1/2} = 3\ 10^{-4}$ $J{\cdot}m^{-2}$, where A is the exchange constant, typically $10^{-11}$ $J{\cdot}m^{-1}$, and K is the anisotropy constant, about $10^4$ $J{\cdot}m^{-3}$. For this case, $L_D$ is of the order of 1 nm.

A different important typical magnetic length is the so-called domain wall width or exchange correlation length, $L_e$. It is usually considered that:

$$L_e = \left(\frac{A}{K}\right)^{1/2} \quad (6)$$

$L_e$ becomes 18 nm for Fe, 5 nm for Ni and Co, 1.3 nm for NdFeB, and 1.2 nm for $SmCo_5$.

It is obvious that NPs with size smaller than either $L_e$ or $L_D$ do not have the volume required to present a domain wall or a domain structure, respectively. Consequently, if they are ferromagnetic, they should be single domains.

Single domains at the low-temperature limit, with frozen magnetic moment, should exhibit peculiar magnetization processes for switching magnetization direction. Coherent rotation and, in general, noncoherent modes have been invoked from micromagnetic arguments and calculations[15].

### Surface Anisotropy and Superparamagnetism in Nanoparticles

Single domain with uniaxial anisotropy constant K and volume V is expected to show fluctuations in the magnetization direction by thermal excitation. At temperatures T, for which KV is $25K_BT$, the magnetization direction reverses by thermal disorder in a few minutes. By assuming a first-order kinetics, with $\tau_0 = 10^{-10}$ sec, we can quantitatively define a blocking temperature $T_B$ as that for which remanence relaxes in 3 min as:

$$T_B = \frac{K \cdot V}{25 K_B} \quad (7)$$

The magnetization for a given T should be observed either as fixed or fluctuating depending on the characteristic time of the experimental technique used for measuring it. A few minutes is the typical time required to measure the remanence of an assembly of NPs by magnetometric techniques such as SQUID. At T above $T_B$, the magnetization behaves as that of a paramagnetic sample and is a function of the ratio H/T, between the applied field H and temperature. For T below $T_B$, the magnetization is blocked and only the effect of either the applied field or quantum tunneling could give rise to magnetization reversal rather than thermal motion[16]. It is important to remark that superparamagnetism defines a limit for decreasing size of NPs to be used as memory elements. Above $T_B$, the NP remanence relaxes to zero in a few minutes. Therefore, such NP systems cannot be used as magnetic recording medium. An estimation of the volume of Fe NPs, for which $T_B$ is the room temperature 300 K, can be obtained from José-Yacamán et al.[7] by using the bulk anisotropy constant of bcc Fe, K = $10^4 Jm^{-3}$. The resultant volume is $10^{-23}$ $m^3$ that corresponds to L = 28 nm. It is important to take into account that if the size of the NPs increases from 28 to 36 nm, the relaxation time (as due to its exponential dependence) should change from 128 to $10^{14}$ sec.

For very small NPs with a high $n_s/n_v$ ratio, it becomes interesting to know which is the anisotropy constant K to be properly included in expression (7). Magnetic anisotropy is a local concept that rises from the combination of the spin-orbit coupling in the magnetic atom and the lack of spherical symmetry in the electric charge distribution of its environment. All the atoms located at the surface undergo a local electric field due to the neighboring atoms with symmetry different to that of the bulk. In fact, one-half of the coordination sphere is different to that characteristic of the internal atoms. This difference in symmetry, together with the difference in spatial charge distribution associated with surface states, leads to a different value of the anisotropy, known as surface anisotropy. Therefore, in NPs, the local anisotropy constant must be an average of bulk and surface anisotropy. The larger deviation from the spherical symmetry corresponding to the surface atoms tends generally to enhance the surface anisotropy constant with respect to that of the bulk.

The more exciting observation of anisotropy enhancement was done by Gambardella et al.[17] that measured, by circular dichroism, the magnetization curve of a single Co atom deposited onto a Pt substrate along both parallel and perpendicular direction to the Pt film. At 5 K, they observed a clear anisotropy indicating a blocked atomic magnetic moment. The giant value of the anisotropy constant required to block an isolated atomic moment is higher than $10^8$ J·m$^{-3}$ or 10 meV per atom. These values can be reached as a consequence of the high strength of the spin-orbit coupling in metals with high atomic weight as is the case of Pt and Au (1.5 eV). Recently, orbital magnetism achieved remarkable importance[18,19,20,21]. Note that when the magnetic moment is originated by the orbital angular momentum, the anisotropy can be very high without involving spin-orbit coupling. Since the angular momentum describes the symmetry of the electric charge distribution, any rotation of the magnetic moment leads to a rotation of the charge distribution. The energy required to rotate the charge distribution that coincides with the anisotropy energy eventually can be high due to direct electrostatic interactions with the local electric field.

### PREPARATION TECHNIQUES

In the following, we present a survey of different methods available to develop metallic NPs, stating the main advantages and limitations of each technique (the methods to obtain magnetic NPs are not different to those for metallic NPs in general). Although these are not the only methods to develop NPs, they are the most commonly used.

#### Chemical Synthesis

Chemical methods are probably the best established for the development of NPs[5,22,23,24,25,26,27, 28,29,30,31,32,33,34]. In general, these methods promote the precipitation of a nuclei constituted by few metallic atoms and subsequent growth. If the growing process is well controlled, it is possible to obtain a narrow size distribution of monodispersed NPs[23,26,27]. The general procedure is the solution of salts containing the specific metal. In the presence of a liquid with an appropriate pH, the salts decompose, liberating the metallic ions. Those ions tend to join, forming particles, and if the growth of such particles is blocked when they are small in the nanometer size range, metallic NPs are obtained. In order to block the particle growth, these methods usually include capping agents that bond to the metallic atoms and prevent them from joining other metallic atoms. The size of the NP can be tuned by controlling the ratio of metallic atoms to capping species in the initial precursors[35]. While in some cases the capping agent just passivates the NP, avoiding further growth, in others, they strongly interact with the NP surface atoms modifying the electronic structure and, therefore, their properties. As the NP has a large fraction of atoms at the surface, the capping agents can strongly modify the properties of the whole NP[10,35,36]. Consequently, it is meaningless to discuss the properties of the NP irrespective of the capping layer. It is the whole nanoentity formed by the NP and the capping layer that stands the properties.

As the metal concentration must be very low to avoid the nucleation and growth of large NPs, the final product is usually in the order of milligrams. However, there are some methods that allow us to prepare large quantities of NPs[31,37].

### Sol-Gel Route

Sol-gel is a particular chemical technique for processing inorganic glasses and ceramics at very low temperatures[38,39,40]. However, if controlled quantities of metallic elements are included in the initial precursors, they can diffuse along the glass and form NPs inside the solid matrix. Thus, the method is suitable to develop magnetic NPs into a solid matrix[41,42,43,44,45,46,47,48]. As the matrix makes the diffusion of metallic atoms difficult to nucleate and grow NPs, the sol-gel route allows us to control the particle size quite accurately, besides giving a material support for the NPs.

Fig. 5 gives a survey of this technique. Initially the precursors — chemical species containing the final constituent materials — are mixed. For silica glassy matrices, the typical precursors are tetraethylorothosilicate (TEOS), tetramethylorothosilicate (TMOS), and methyl-trietoxisylane (M3ES). In the presence of a solution with an appropriate pH, the precursors are hydrolyzed, breaking into smaller units. Silica precursors usually form $Si(OH)_4$ groups, while the salt used as precursor of the metallic atoms breaks into a metallic ion plus cations. For instance, iron nitride that is used as precursor for Fe, follows the reaction $Fe(NO_3)_3 \rightarrow Fe^{3+} + 3NO_3$[48]. Subsequently, the small $Si(OH)_4$ groups start to join each other (polycondensation), forming structures of nanometric size, which is called *sol*. In this step, the metallic ions join the network by means of oxygen bonds. In the gelation step, those nanostructures grow to form a bulk network, while the system loses water content, the viscosity increases and the system passes to the solid state. The system is aged for a few days while polycondensation continues and the porosity of the material is reduced. The gel is then dried at room temperature (or slightly above) to reduce the water content. Finally, the material is heated in order to remove the water and the rest of organic matter entrapped into the pores. This is an optional step that determines the density of the material and, therefore, the mechanical consistence. Depending on the metal and fabrication parameters, the NPs can be formed before heating inside the glassy matrix. Otherwise, their nucleation and growth can be induced by annealing, which favors the diffusion of metallic atoms along the matrix to join another atoms and create NPs. The temperature, time, and atmosphere of the treatment control the final size distribution of the NPs. This method allows growth of core-shell NPs[46,47] and also alloys[41,42,43,45,49].

### Ion Implantation

Ion implantation is a well-established technique initially designated to dope materials (mainly semiconductors)[50], but it is also applied successfully to develop metallic NPs into solid matrices[51,52,53,54,55,56,57]. Fig. 6 describes the experimental set-up to perform ion implantation. The technique is based on generating a plasma with the ions to be implanted. The ions are extracted and preaccelerated to select the mass by means of a magnet. Once the beam is free of impurities, it is accelerated to the desired energy and implanted on the substrate. As the ions are implanted, the substrate charges electrically and if it is an insulator, the charge cannot be eliminated via a ground connection. Due to the excess of charge, the electric field can interact with the ion beam, modifying the implantation process. To avoid this problem, some devices have an electron gun that compensates the charge of the ion beam. The dose and energy of the implanted ion will determine the final concentration profile of the implanted species into the solid. If the dose is large enough, NPs are formed inside the substrate. Furthermore, even if the doses are low, subsequent annealing can promote the diffusion of the ions and, therefore, the nucleation of NPs. The main advantage of the technique is that by controlling the implantation parameters (dose and energy) and the subsequent annealing process (temperature, time, and atmosphere), it is possible to tailor the NP composition, size, and spatial distribution. The technique results are particularly interesting for development of alloy NPs[55]. When different species are implanted subsequently, the implantation order can alter the structure of the NP completely[58]. For instance, when FeAl NP are formed by sequential ion implantation, it is found that if the Fe is implanted first, it yields to core-shell structures, while implanting Al before Fe gives rise to bcc Fe-Al alloy NPs[59,60].

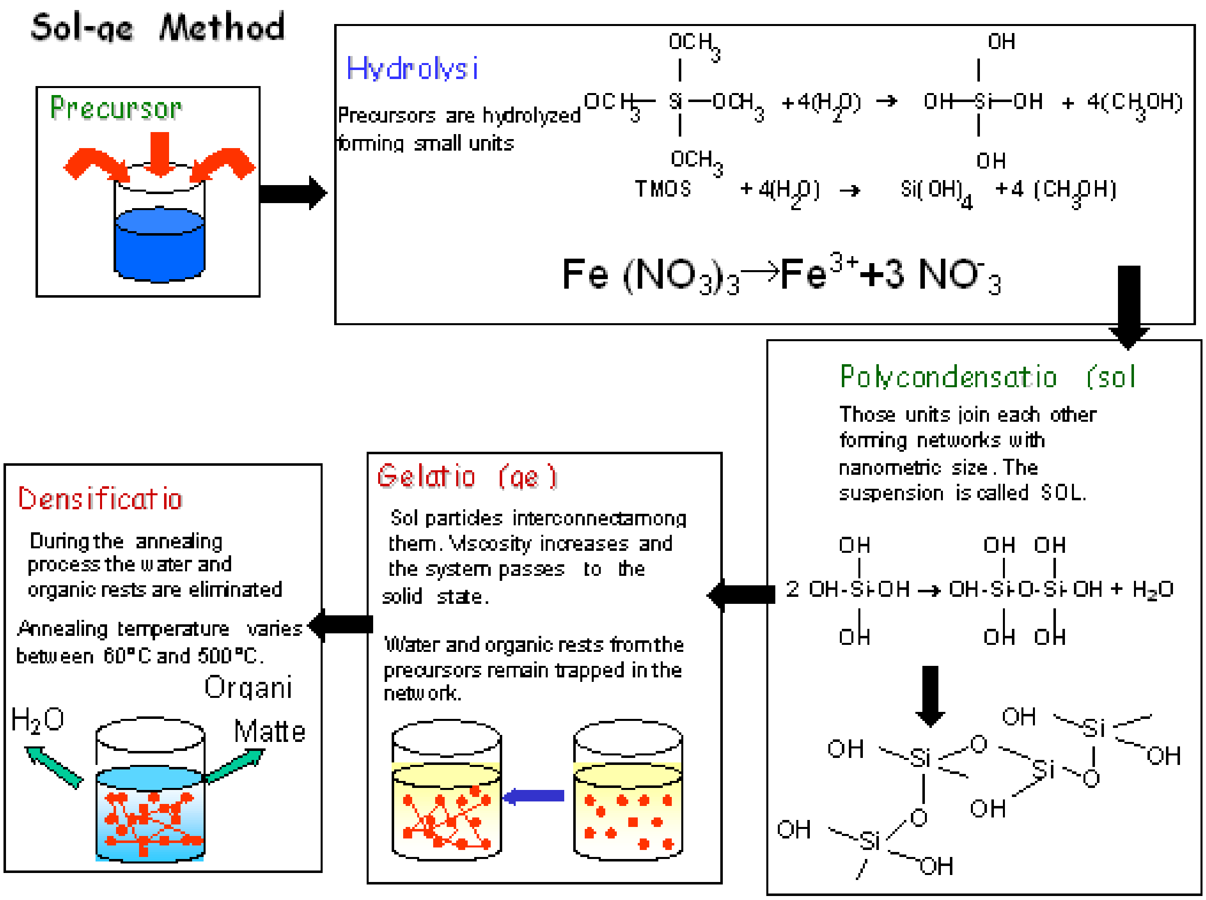

**FIGURE 5.** Survey of the sol-gel method.

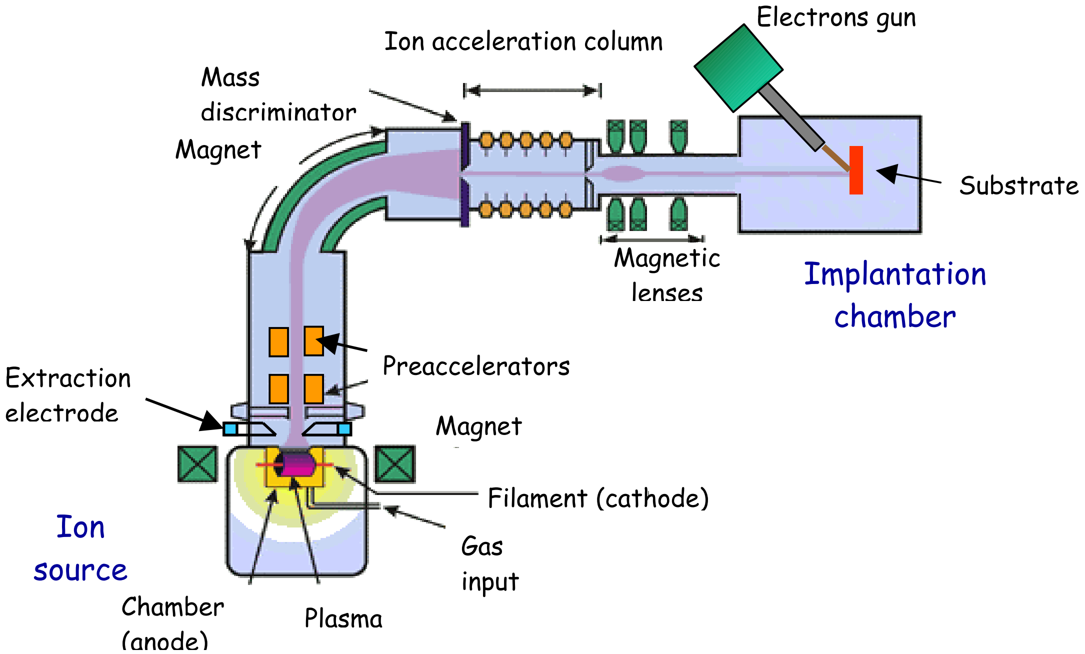

**FIGURE 6.** Scheme of the experimental set-up for ion implantation.

### Amorphous Nanocrystallization

NPs can be obtained from amorphous material (bulk, ribbons, wires, etc.) by controlled nanocrystallization[61,62,63,64,65,66,67,68,69,70,71,72,73]. As the amorphous is a nonequilibrium state, when the material is subjected to thermal treatments, the crystallization of the material takes place. The crystallization process consists of the nucleation of small nanocrystals and subsequent growth up to the percolation. If the crystallization process is stopped in the first stages, before percolation takes place, the result is a distribution of NPs embedded into an amorphous matrix. The temperature, atmosphere, and time of the annealing process allow control of the size distribution of the NPs. NPs obtained by this method usually exhibit anisotropy fairly larger than the amorphous matrix that is magnetically soft. Thus, the coupling of the NPs with the amorphous matrix and with other NPs can give rise to different magnetic behaviors[64,74]. These nanocomposites formed by nanocrystals embedded into an amorphous matrix create very interesting results for the development of sensors and detectors[75]. Obviously, the NPs that can be obtained by this method are restricted to those compositions for which it is possible to obtain amorphous metals, mainly FeB-based alloys.

### Film Deposition Techniques

Film deposition techniques such as sputtering, plasma laser deposition, or evaporation can also be used to fabricate NPs[76,77,78,79,80,81,82,83]. The most common technique to obtain the NPs consists of depositing continuous thin films and promoting nanocrystallization processes in a way similar to that of the amorphous nanocrystallization process described above. In this case, the results are nanocrystals embedded in an amorphous film. If a film with a composition different to NPs is desired, it can be achieved by depositing multilayers or by codeposition processes. For instance, FePt NPs embedded into Ag matrices can be obtained by depositing multilayers of FePt/Ag and annealing at low temperatures[77,84]. Similarly, Fe, Co, or Ni NPs into silica matrix have been prepared by codeposition of the metal and the silica[85].

If the deposited dose is below one monolayer, the metallic atoms tend to diffuse on the surface of the substrate, forming islands of nanometric size which are a kind of NP[86,87,88]. The temperature and atmosphere during deposition, as well as metal substrate interaction, will govern the diffusion on the substrate surface.

### High-Energy Ball Milling

In recent years, high-energy ball milling and mechanical alloying have emerged as alternative synthesis techniques for phases far from equilibrium conditions. Both can be considered as solid-state powder processing techniques involving repeated welding, fracture, and rewelding of powder particles in a high-energy ball mill. The process is carried out in a vial where blended elemental or prealloyed powders are introduced together with a single or several balls. The vial is then subjected to a specific movement depending on the type of milling device. The energy of the process depends on several factors such as the type of milling device, milling container, amplitude of vibration, rotational speed, and ball-to-powder ratio, among others. In all cases, the main event that occurs is the ball-powder-ball collision. Powder particles are trapped between the colliding balls and undergo deformation, cold welding, and fracture processes that define the final structure of the powder. The nature of the processes depends on the mechanical behavior of the powder components, their equilibrium phases, and the induced stresses by milling. A review on mechanical alloying and milling can be found in Suryanarayana[89]. Although it was developed to produce oxide-dispersion strengthened (ODS) alloys by Benjamin and co-workers in the late 1960s[90], the process attracted new interest in the early 1980s since it was discovered that solid-state amorphization can be induced by high-energy ball milling[91]. Since then, it has been shown as a powerful synthesis method that allows us to obtain a wide variety of equilibrium and nonequilibrium phases, including metastable crystalline and quasicrystalline phases, amorphous alloys, supersaturated nanocrystalline solid solutions, and nanostructures[89,92,93].

As will be shown, one of the main aspects that occurs on milling is the refinement of the microstructure of the starting materials up to the nanoscale regime. This process modifies the free energy curves of the system with respect to the equilibrium conditions. In some cases, it allows the stabilization of nanostructures as occurs in the case of nanocrystalline Pd. As an example, Fig. 7 shows the evolution on milling of high-purity Pd powders. Agatha vials and balls were used to prevent contamination coming from the milling media. The peaks corresponding to face-centered cubic Pd become broader as the milling time is increased, indicating a refinement of the crystallite size and an increase of the atomic level strain induced by milling. In the case of Pd, the average crystallite size achieved by milling is around 19 nm. Fig. 8 shows a TEM micrograph of the particles obtained after 6 h of milling. Nanocrystals with average sizes between 20 and 25 nm are clearly observed in the micrograph.

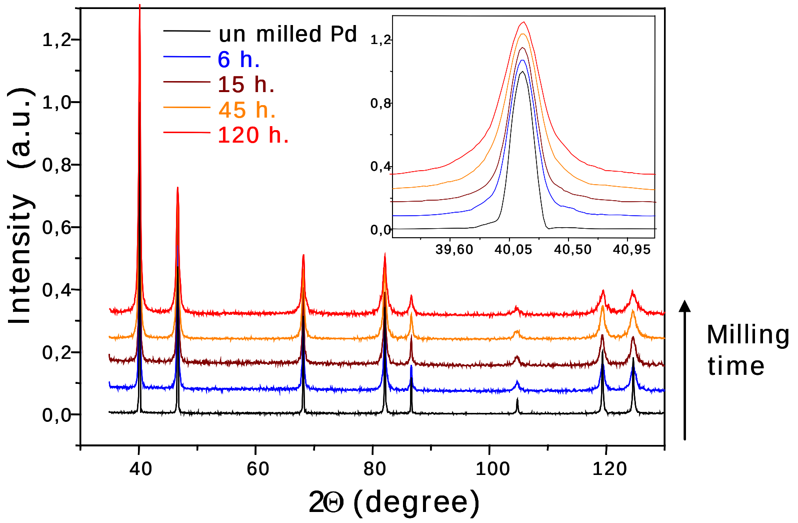

**FIGURE 7.** XRD pattern from Pd powder milled at different times showing the decrease of particle size.

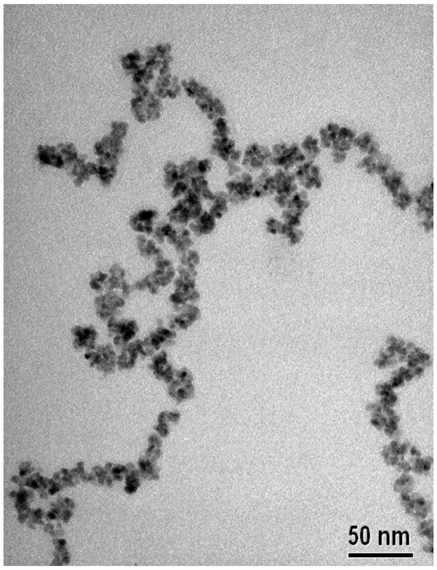

**FIGURE 8**. TEM micrograph corresponding to Pd powder milled for 6 h (courtesy of B. Sampedro).

Eckert et al. have proposed that the minimum grain size achievable by milling is determined by the minimum grain size that can sustain a dislocation pile-up within a grain. Thus, the grain size of the NPs saturates at a steady-state value and, even with prolonged milling, no further refinement of the grain size occurs[94].

Although high-energy ball milling has some limitations concerning the fabrication of nanostructures with particle sizes below 10 nm, it has an enormous advantage when considering the possibility of obtaining new nanocrystalline magnetic systems; in particular, solid solutions of immiscible elements.

As mentioned previously, in most systems, the refinement of the microstructure produces a change of the shape of the Gibbs free energy curves with respect to that of the equilibrium conditions, thus allowing the synthesis of nonequilibrium phases. For instance, this is what occurs in the case of immiscible elements such as Fe and Cu, or Cu-Co. The Fe-Cu system is a phase-separating system characterized by a high and positive enthalpy of mixing. Fig. 9 shows a diagram of the free energy curves for the Fe-Cu system[95]. The free energy curves indicate that both the fcc and bcc solid solutions are in a higher energy state as compared with a mixture of fcc-Cu and bcc-Fe phases. It was proposed that the alloying is only possible when the intense deformation generates nanometer-size fragments of bcc-Fe. This produces an increase of the free energy of the fcc-Cu and bcc-Fe mixture due to the increase of the surface energy in such a way that the alloy formation is favorable when the bcc-Fe fragments decrease down to a 2-nm size. It has been reported that mechanical alloying allows the synthesis of nanocrystalline Fe-Cu solid solutions in all the compositional range. The alloys exhibit a face-centered cubic structure when the Fe content is below 60 at.%, and a body centered structure when the Fe content is above 70 at.%. It should be also noticed that the alloying promotes ferromagnetism in the fcc-Fe-Cu alloys although fcc-Fe and fcc-Cu are not ferromagnetic at ground state. A review on Fe-Cu solid solutions can be found in Crespo and Hernando[96].

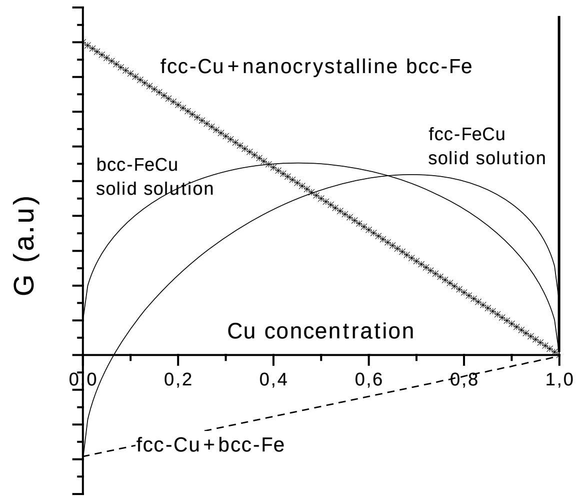

**FIGURE 9.** Diagram of the free energy curves for the Fe-Cu system.

### CHARACTERIZATION TECHNIQUES

Characterization techniques for NPs (both structural and magnetic) are mainly those used in general in materials science and are too extensive to be reviewed here. In the following, we describe some techniques whose results are particularly suitable for the study of NPs, paying particular attention to the information that can be achieved by means of these techniques.

### Surface Plasmon Resonance

Interaction of light with NPs creates results that are very interesting for the analysis of the NP because it is largely dependent on the particle size and reflects the changes in the electronic structure of the NP with the number of atoms.

Surface Plasmon Resonance (SPR) is one of the most outstanding optical properties of metallic NPs[97,98,99]. SPR consists of a collective oscillation of the conduction electron inside the NP. The excess of charge produced at the surface because of electron movement acts as a restoring force (see Fig. 10), while the electron movement is damped mainly because of the electron interaction with atomic cores and NP surface.

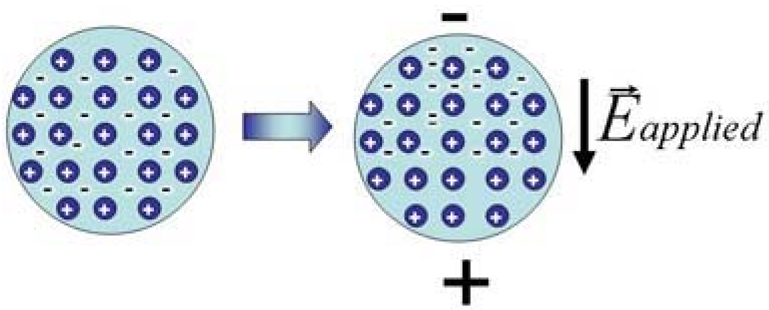

**FIGURE 10.** SPR of metallic NPs. When the light passes through the NP, the electric field makes the electrons oscillate inside the NP. The excess of charge creates an electric field opposite to that applied.

The system acts as a damped oscillator with a resonance frequency that, for most of the transition metals, lies on the UV-Vis part of the spectrum. Hence, the NP exhibits an absorption band in this region of the optical absorption spectrum. As the damping depends strongly on the particle size, the shape of the SRP also does as Fig. 11 shows. Therefore, the analysis of SPR provides information about the particle size and electronic configuration of the NP. The presence of SPR is a fingerprint of free electrons inside the NP (and, therefore, of its metallic character), while the absence indicates the localization of electrons. Recent measurement on Au NP capped with different molecular species showed that certain capping agents yield to charge localization at the NP surface and, therefore, the SPR disappears from the spectrum as shown in Fig. 11.

Besides the SPR, interband transitions absorption also provides information about the electronic structure of the NP. As Fig. 1 shows, metallic NP presents a states distribution that is intermediate between single atoms and bulk materials; there are some energy bands, but these are not large enough to overlap. Thus, it is possible to excite electrons from one band to another by absorption of one photon. Therefore, interband transitions will give rise to a border in the optical absorption spectrum. Depending on the particular metal, this border can fall on the SPR band or be separated.

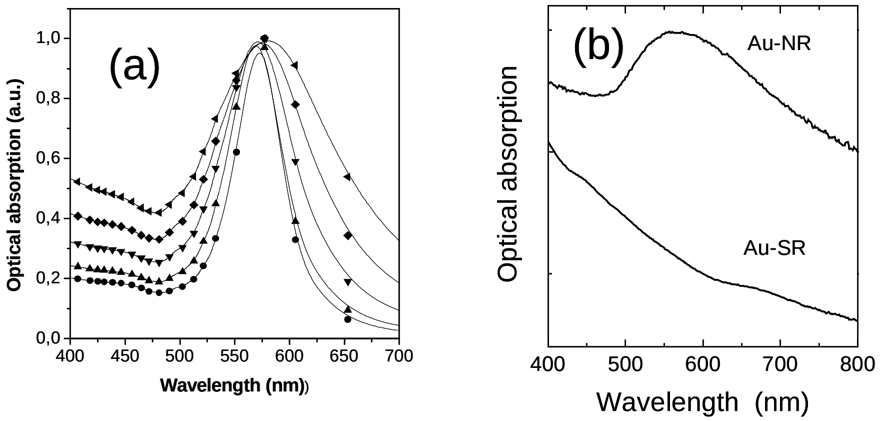

**FIGURE 11**. (a) SPR from Au NPs with R = 2 nm (◄), 3 nm (◆), 5 nm (▼), 10 nm (▲), and 20 nm (•); (b) SPR from Au NP capped with a surfactant (top) and with thiols (bottom)[10,99].

### X-ray Techniques

#### *X-ray Diffraction*

X-Ray Diffraction (XRD) and absorption experiments are commonly used in materials science as these techniques provide information about the size, structure, and electronic distribution of materials[67,69,100].

While diffraction peaks are about delta functions for bulk crystalline materials, for small NPs, the peaks are broad. The width of the peaks increases as the nanocrystal size decreases, so it is possible to measure the average size from the XRD patterns using the Debye-Scherrer equation[69].

The small size of the NP and the low crystallinity of the samples imply a low signal when interacting with X-rays[101]. Thus, large intensities of X-rays are desired in order to get good signal-to-noise ratio in the measurements. Therefore, synchrotron radiation is very suitable both for diffraction and absorption experiments, in particular for NPs below 4-nm size. For these sizes, the large intensities of synchrotron radiation allow spectra collection in a few hours that should take several weeks in a conventional X-ray diffractometer. In addition to being able to check the existence of small nanocrystals, XRD experiments are interesting in order to analyze the size effects on the crystal structure of the NPs. In general, small NPs (below 5-nm size) exhibit a larger lattice parameter than the corresponding bulk materials, in order to reduce the surface energy which is comparable to the energy volume at these sizes. As the surface energy will obviously depend on the capping species, the lattice expansion depends not only on the particle size, but also on this capping agent. The large intensity of synchrotron radiation allows us to obtain low-noise diffraction spectra that permit us to calculate the lattice parameter very accurately and, therefore, to determine small changes with respect to bulk materials. As an example, small Au NPs exhibit a significant contraction in the lattice parameters with respect to bulk Au because of size and surface effects[36,102,103]. However, when the NPs are capped with thiols, the effect is not so pronounced.

#### *X-ray Absorption Spectroscopies*

Another advantage of synchrotron radiation is the large spectrum of X-rays, which allows us to perform spectroscopy. The X-ray absorption experiments also provide interesting information about the electronic configuration of the NPs. In particular, X-ray Absorption Near Edge Spectroscopy (XANES) gives information about the population around the Fermi level. As an example, Fig. 12 shows the XANES spectra for Au NP capped with different capping agents[10]. The sample is illuminated with X-rays in the energy range close to the energy difference between two electronic levels, in this case, the 2p and 5d levels. Electrons at the 2p level can absorb a photon and pass to the 5d level. The rate of transitions (and, therefore, the intensity of X-ray absorption) will depend on the number of empty states in this later orbital. Therefore, XANES spectra give information about the electronic structure of the samples. While for the sample named Au-NR (Au NPs capped with tetraalkylammonium) the electronic configuration is similar to that of bulk Au, the sample Au-SR (Au NPs capped with thiols) presents and enhanced absorption ascribed to a higher density of empty states at the 5d orbital. This difference is due to the charge transfer from Au to S atoms at the end of the thiol chain.

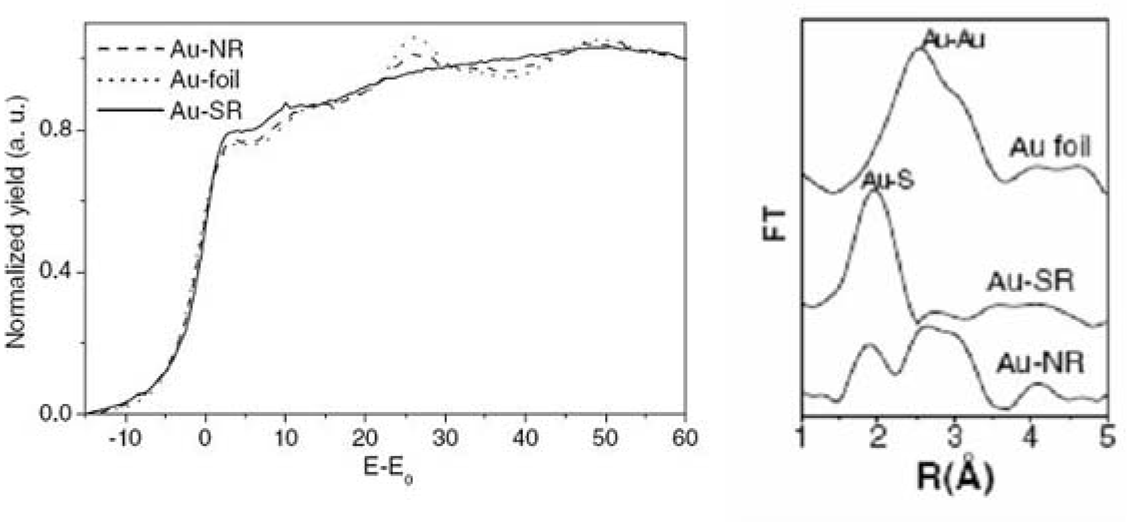

**FIGURE 12.** (Left) XANES spectra of Au NP capped with two different molecules. The spectra of foil Au is also presented for comparison. (Right) EXAFS spectra corresponding to the same samples. From Ref. [10].

Another interesting technique based on X-ray absorption experiments is the Extended X-ray Fine Absorption Structure (EXAFS). The analysis of the X-ray absorption spectra close to the absorption edge permits, by means of a Fourier Transform, determination of the interatomic distances, identifying bonds present in the NP and, therefore, giving valuable information about its structure. This technique can be very useful to analyze possible segregation of a certain element or the formation of alloys inside the NP. In the case of Au NPs capped with thiols, the EXAFS spectra show the presence of Au atoms bonded to the S atoms at the end of thiol chains (see Fig. 12).

### *X-ray Photoelectron Spectroscopy*

X-ray Photoelectron Spectroscopy (XPS) is a particular X-ray absorption technique that does not require synchrotron radiation since it can be performed with monochromatic X-rays and the results are very useful to analyze magnetic NPs[104,105,106,107,108,109,110]. The principles of the technique are shown in Fig. 13. The sample is illuminated with monochromatic X-rays energetic enough to pull out electrons from the atom. When an electron absorbs a photon, it leaves the atom with a kinetic energy $E_k = E_\gamma - E_0$ with $E_\gamma$ being the photon energy and $E_0$ the energy corresponding to the original orbital of the electron. As the electronic levels in the atoms are discrete, we found peaks in the energy spectra of photoemited electrons. Analyzing those peaks, we obtain information about the orbital energy, i.e., information about the energy levels of the atom. Since these energy levels are characteristic of each atom, the technique allows us to detect the presence of particular elements. Moreover, the outer energy levels of the atoms are modified by the crystalline field in the presence of other atoms, which turns into shift of the XPS peaks. Therefore, it is possible to determine the environment of the atom, ionizing states, and particular bonds to other atoms. For instance, $Fe^{2+}$ and $Fe^{3+}$ can be identified separately by means of XPS[104,105]. Similarly, Co NPs with bcc or fcc structure can be distinguished by means of this technique[106]. In the case of NPs with a large fraction of the atoms at the surface, XPS measurements can be used to study the interaction of capping agents[107,109]. A recent work on Ni NPs showed how capping with silica alters the electronic structure of the samples[107].

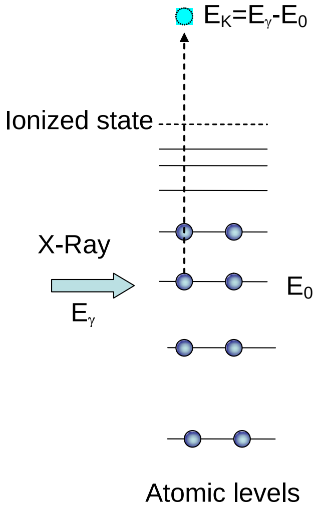

**FIGURE 13.** Scheme of XPS process.

### X-ray Circular Magnetic Dichroism

X-ray Circular Magnetic Dichroism (XCMD) is a magnetometric technique that is particularly interesting for the analysis of NPs[17,86,87,88,111,112,113,114,115]. Usually, NPs are embedded in a medium or deposited onto a substrate in which they only represent a small percentage of the total mass. Thus, the presence of impurities in the surrounding media can lead to large mistakes in the measurement of their magnetic properties.

The XCMD is based on the fact that the absorption of light by the matter may depend on the relative light polarization and the spin orientation of the atomic magnetic moments. Thus, when an electron has a certain polarization of the spin, the absorption of light at a certain edge will be different for light right-handed polarized or left-handed polarized, as Fig. 14 illustrates. Actually, measuring the absorption of both light polarizations, it is possible to calculate the magnetic moment of the electron by means of the so-called summation rules. Furthermore, the technique allows us to separate the spin and orbital contribution to the magnetic moment.

As the measurements are carried out at the absorption edge of a certain element, we only observe the contribution of this particular element. This feature is particularly interesting when dealing with alloy NPs, as it allows us to measure separately the contribution of each element to the total magnetization. For instance, contribution of Fe and Pt to magnetization of Fe-Pt nanostructures has been recently measured separately showing that, surprisingly, the presence of Pt increases the magnetization of Fe[116].

### Electron Microscopy

Nowadays, the most powerful tool to image metallic NPs is Transmission Electron Microscopy (TEM)[117]. In particular, High Resolution Electron Microscopy (HREM) provides accurate data about the size, shape, and crystallography of the NP, which cannot be achieved by any other technique[7,9,10,118]. HREM has atomic resolution that allows us to determine the crystallographic structure and lattice parameter of very small NPs, as well as the presence of defects (see Fig. 15). An interesting mode of TEM is the Selected Area Electron Diffraction (SAED). While XRD gives average information on the sample, SAED allows us to obtain the diffraction pattern from a single NP and, therefore, it presents the possibility for studying changes in the crystallography for particles with different size. Fourier Fast Transform is also a powerful tool to analyze the HREM images with excellent results[9,10].

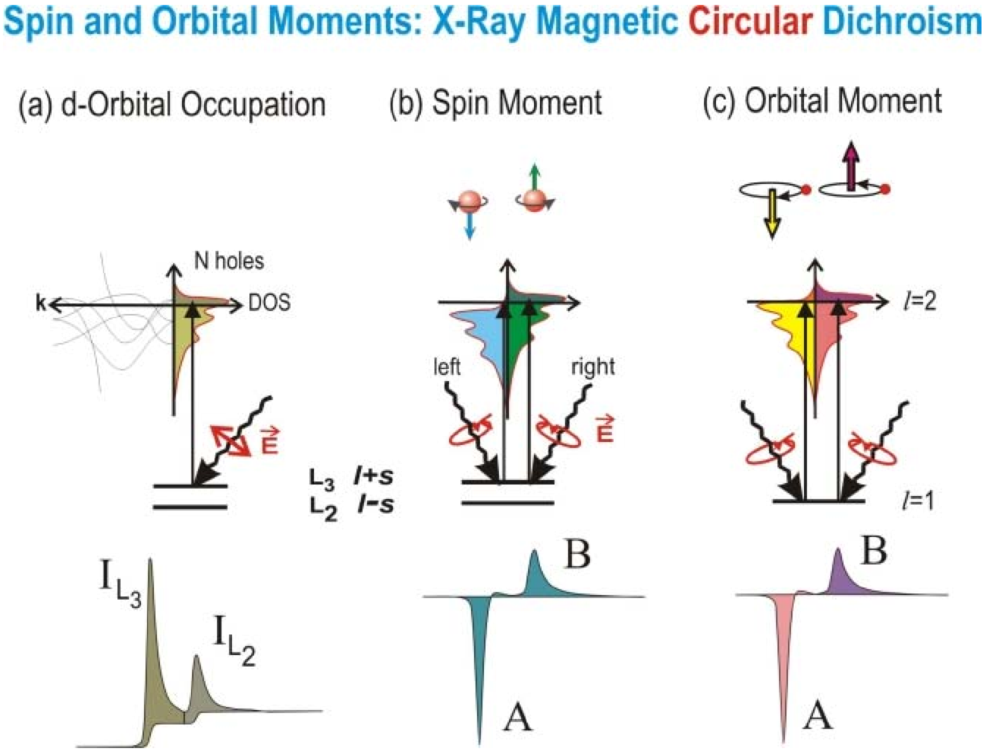

**FIGURE 14**. Scheme of the principles of XMCD. From ref. [112]. Reproduced with permission of the author.

### Scanning Probe Microscopies

Scanning Probe Microscopies (SPM) are powerful techniques that are complementary to TEM measurements[119]. While the TEM gives information about the total volume of the NP, SPM deal only with the surface. The main advantage of the SPM is that they are noninvasive techniques that do not modify the nanostructures, as can happen with the energetic electronic beam of TEM. Those SPM techniques scan the surface of the sample with a tip that is moved by means of piezoelectricity and measure different kinds of interactions between the tip and the sample. Scanning Tunneling Microscopy (STM) measures the tunnel current between the sample to the tip that depends on their proximity and on the electronic configuration of the sample. STM has atomic resolution and gives information not only on the topography of the sample, but also on the electronic density of the sample. However, STM is limited to conducting samples and it is hard to obtain information from insulating samples that must be covered with a layer of conducting material to perform the measurements. Atomic Force Microscopy (AFM) is another kind of SPM. In the dynamic modes[120], the tip is initially forced to oscillate at its resonant frequency by means of piezoelectricity. When the tip approaches the sample, there are changes in the tip oscillation (amplitude resonant frequency, etc.) due to tip-sample interactions, which are measured by using a laser and a photodiode as Fig. 16 illustrates. As the tip scans over the sample surface, we get a topographic image of the sample. It is a noninvasive technique with a lower resolution than SPM, but it is possible to observe even a 1-nm size NP, and there is no limitation on the nature of the NP[33,121,122,123,124]. As an example, Fig. 17 shows AFM images of small metallic NPs onto a mica substrate[125]. In any case, AFM measurements of small NPs must be carried out carefully as the observation of these NPs is not straightforward and can lead to large mistakes in sizing them. In particular, while the NP height measured by AFM is quite accurate, the width is largely distorted by the convolution with the tip geometry.

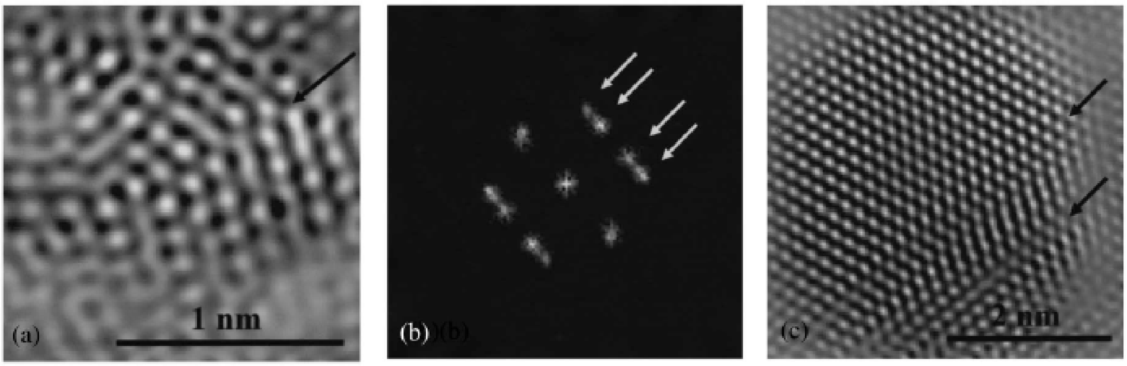

**FIGURE 15.** (a) Fourier-filtered HRTEM image of a Pd NP (diameter ~ 2 nm). The twin boundary is arrowed; (b) corresponding FT diffraction pattern along the [111] crystallographic direction. The splitting of the FT spots due to twinning is indicated; (c) Fourier-filtered HRTEM image of a Pd nanocrystal (diameter ~ 4:4 nm) showing two twin boundaries. From ref. [9].

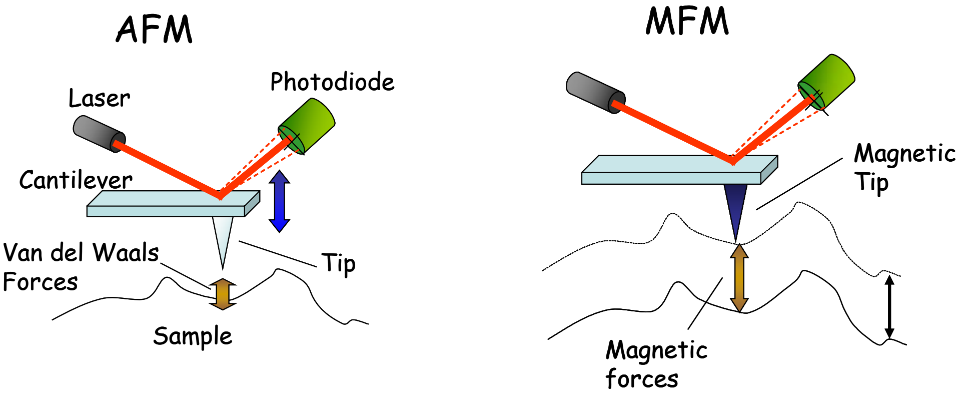

**FIGURE 16**. Scheme of AFM (left) and MFM (right) measurements process.

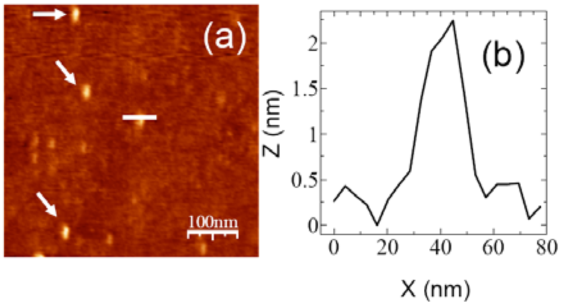

**FIGURE 17.** (a) AFM images of 2.4-nm Pd NPs onto a mica substrate; (b) height profile along the indicated line. (Courtesy of I. Carabias.)

SPM results are complementary to TEM measurements. While TEM only sees the metallic core of the NP, the SPM measures the whole ensemble formed by the NP and the capping layer, so both measurements can give information about the structure of the nanoentity.

A particular working mode of the AFM is Magnetic Force Microscopy (MFM)[114,119]. For these measurements, the AFM tip is capped with a magnetic material. Thus, beside the atomic forces, there is a magnetic interaction between the NP and the tip[126,127,128,129,130]. Different than the Van der Waals interactions, the magnetic forces are of large range and, therefore, the dominant choice for distances larger than tens of nanometers. To obtain information about the magnetic state of the sample, the procedure is as follows (described in Fig. 16): initially a scan is performed on the sample with the tip very close to the sample. In this situation, the atomic forces are larger than the magnetic ones that can be neglected and we obtain a pure topographic profile of the sample. Subsequently, we move the tip up (distance about 100 nm) and we repeat the previous scan profile. In this mode, the tip is always at the same distance to the sample and there is no topographic contrast, so we get magnetic contrast.

The main limitation of the MFM measurements is that they require a large magnetic signal. As the magnetic measurements are performed at distances around 100 nm and the tip mass is large compared with NP size, if the NP is small or the magnetization is low, it is not possible to observe perturbation in the tip oscillations due to magnetic interactions. Thus, magnetic contrast in NPs is achieved only for NPs with high saturation magnetization and sizes of tens of nanometers.

### RECENT EXPERIMENTAL RESULTS

As explained above, the nanoscale alters the properties of bulk ferromagnetic materials that, in the form of NPs, show different coercivity, remanence, relaxation time, etc. However, this is not the only effect of nanoscale on the magnetic properties of the materials. Recent experimental results showed that the nanoscale can also induce ferromagnetic behavior in materials that are nonferromagnetic in bulk state.

In particular, it has been demonstrated that small Pd NPs show ferromagnetic behavior[9,131] as shown in Fig. 18. Pd is an fcc metal exhibiting Pauli-enhanced paramagnetism and close to ferromagnetic instability. Although Pd has a large density of states at the Fermi level, ($N(E_F) = 1.23$ $eV^{-1}$ $spin^{-1}\cdot atom^{-1}$), it is not enough to satisfy the Stoner criterion for ferromagnetism (for Pd $J = 0.71$ eV and, therefore, $N(E_F)\cdot J = 0.87 < 1$). Surprisingly, in this NP, the magnetic behavior is almost independent of T in the range 5–300 K, without superparamagnetic effects, as it happens for NPs of bulk ferromagnetic materials. The detailed analysis performed by TEM on these NPs (see Fig. 15) showed that most of these NPs exhibit twin boundaries in order to reduce their energy; fcc metals present a highly anisotropic surface energy. In a spherical NP, all the possible surface orientations are present. However, the presence of twin boundaries allows us to avoid the most energetic orientations, therefore reducing the surface energy. The twin boundary formation energy for fcc metals is quite low and, thus, in the energy balance, these twin boundaries reduce the total (surface plus volume) energy. In this situation, the NPs exhibit a large fraction of atoms without cubic symmetry: those at the surface (representing a significant percentage of the total) and those close to the twin boundaries. The lack of cubic symmetry prevents the splitting of d band into the $t_{2g}$ and $e_g$ sublevels and, therefore, it yields to narrowing of the band. Hence, in the vicinity of those atoms, there is a higher density of states that could be enough to satisfy the Stoner criterion.

More astonishing is the case of Au NPs that also exhibit ferromagnetic behavior up to room temperature[10]. Differently to the case of Pd, Au is diamagnetic with a very low density of states at the Fermi level ($N(E_F) = 0.3\ eV^{-1} \cdot spin^{-1}\ atom^{-1}$). For Au NPs, the presence of ferromagnetic behavior is associated to selective capping of the NP with certain molecules. Au NPs capped with weak interacting tetraalkylammonium that merely passivate the particle and avoid further growth (named Au-NR) show diamagnetic behavior as bulk Au does (Fig. 19). On the contrary, Au NPs capped with strongly interacting thiols (named Ay-SR) exhibit ferromagnetism up to room temperature as shown in Fig. 19. In this case, the presence of ferromagnetic behavior is associated with the covalent bond between Au atoms at the NP surface and the S atoms at the end of the thiol chain. This bond gives rise to a charge transfer from the Au to the S (see “X-ray Absorption Spectroscopies” and Fig. 12), therefore modifying the electronic configuration of both atoms and, consequently, the value of their magnetic moments.

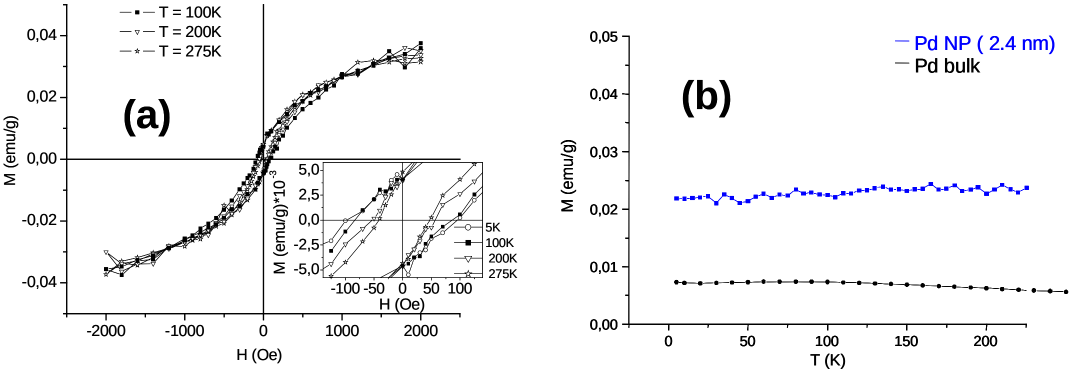

**FIGURE 18.** (a) Hysteresis loops of 2.4-nm Pd NPs; (b) thermal dependence of the magnetization (data from bulk Pd are also shown for comparison). From ref. [9].

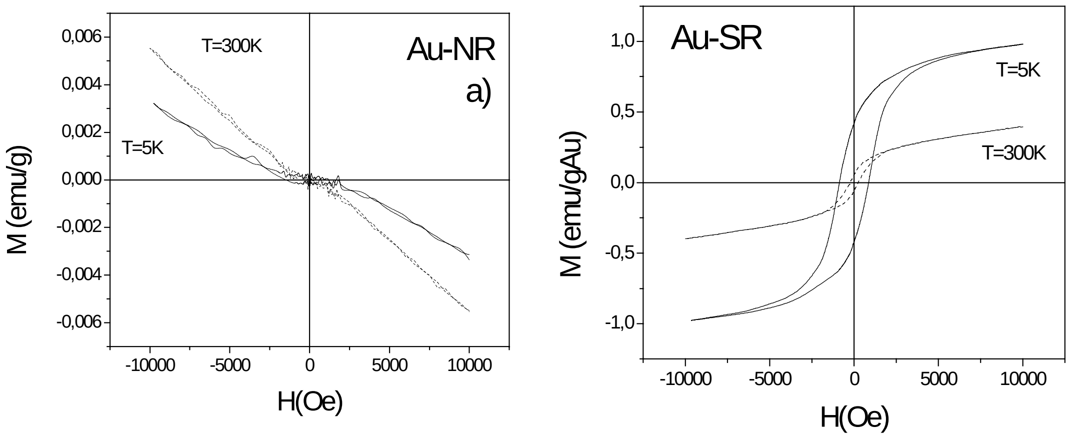

**FIGURE 19**. Hysteresis loops from 1.4-n Au NPs capped with (left) tetraalkylammonium and (right) thiols. From ref. [10].

Experiments carried out on Au thin film capped with thiols confirmed that magnetism is associated to the bond[11]. In this case, ferromagnetic behavior is observed when the field is applied perpendicular to the film (i.e., parallel to the bond) whereas a very weak signal is found when the field is applied parallel to the film.

Although the ferromagnetic behavior of Pd and Au NPs has different origin, they present a common feature: the presence of ferromagnetic behavior up to room temperature without superparamagnetic effects and this is a fingerprint of a huge anisotropy. In the case of Pd, such anisotropy can arise from the lack of symmetry already pointed out, that should block the orbital momentum in certain directions. Then, the large spin-orbit coupling should block also the spin in these directions, accounting for the observed anisotropy as explained in “Surface Anisotropy and Superparamagnetism in Nanoparticles”. For the Au NPs, spin-orbit coupling can also be invoked as the origin of the huge anisotropy, but in this case, there are other possibilities. As explained above, the magnetism is associated to the Au-S bond and the magnetic moment can be in both atoms. While the spin-orbit coupling (1.5 eV) for magnetic moments in Au can be responsible for the anisotropy, the magnetic moments hold by the S atom could be orbital from the electron participating in the bond, whose orbital moment is blocked by the bond (with typical energy of few eV).

### BIOMEDICAL APPLICATIONS

Magnetic NPs have a lot of potential applications in many fields that will not be described here. We will summarize some biomedical applications that have received great interest in the last years as an example of cross-disciplines in nanoscience and nanotechnology.

NPs are within the size of biological entities (from few nanometers up to about 100 μm), so they are very interesting for biomedical applications. Those agents are cells (10–100 μm), viruses (20–450 nm), proteins (5–50 nm), or genes (2 nm wide and 10–100 nm long), so magnetic particles with different sizes are needed. Among the different kinds of nanostructures, magnetic NPs (NP) are particularly attractive because they can be remotely controlled via magnetic fields that penetrate easily into organic tissue and act at distance (without contacts). Nowadays, in the frame of biomedicine, there are mainly four different applications of magnetic NPs[5,132,133,134,135] clearly identified:

- **Cell separation** — The particles are capped with molecules that tag to certain target entitites (cells, viruses, genes, etc.) in liquid solutions. Once the target has been reached, the particles are separated via a magnetic field.
- **Drug delivery** — The drugs for several treatments (mainly chemotherapy) are attached to the magnetic NPs and put into the blood vessel. By applying magnetic fields in the region close to the damaged part of the body, those drugs can be confined in this region, enhancing their effectiveness. Besides, it reduces the effect on the rest of the body, allowing the use of more active drugs.
- **Hyperthermia** — Ferromagnetic NPs are capped with molecules that tag to tumor cells when circulating into the cardiovascular system. The application of alternating magnetic fields yields to heat dissipation (proportional to hysteresis loop area) that can selectively kill tumor cells that die at a temperature slightly lower than healthy cells.
- **Magnetic resonance imaging** — In this technique, magnetic NPs are capped with tags to detect certain types of cellular receptors (typically tumor cells)[136,137]. Thus, when the particles are injected into the blood vessel, they circulate until they reach their target, remaining localized at this point. The presence of these particles originates a faster relaxation of the water molecules in the vicinity of the ferromagnetic particle that reduces significantly the intensity of its nuclear magnetic resonance signal and causes darkening in the images.

Fig. 20 summarizes the scheme of some of those processes. The main limitation in the use of magnetic NPs for biomedical applications is the low biocompatibility of classical magnetic materials. Ni and Co are toxic and susceptible to oxidation, so they are overlooked when considering biomedical applications[133,138]. Although the human body is designed to process Fe (in the human body, Fe is stored in the core of protein ferritin), its bioavailability is limited and can be toxic to cells[139]. Thus, the recent discovery of ferromagnetism at room temperature in noble metal NPs (highly biocompatible) can help to overcome this limit.

Hence, currently, magnetic NPs used for biomedical applications are mainly formed by a magnetic core (usually magnetite $Fe_3O_4$ or maghemite $\gamma$-$Fe_2O_3$) with biocompatible coatings (for instance, $SiO_2$) to avoid direct contact of the magnetic nuclei with the biological fluids that could oxidize or dissolve it[5]. Finally, the NPs are capped with appropriate molecules to functionalize them. In some cases, these molecules are also the biocompatible coating. The role of the functional molecules is to tag the NPs and favor that they fix at certain target positions inside the biological tissue: specific cells, genes, and/or chains.

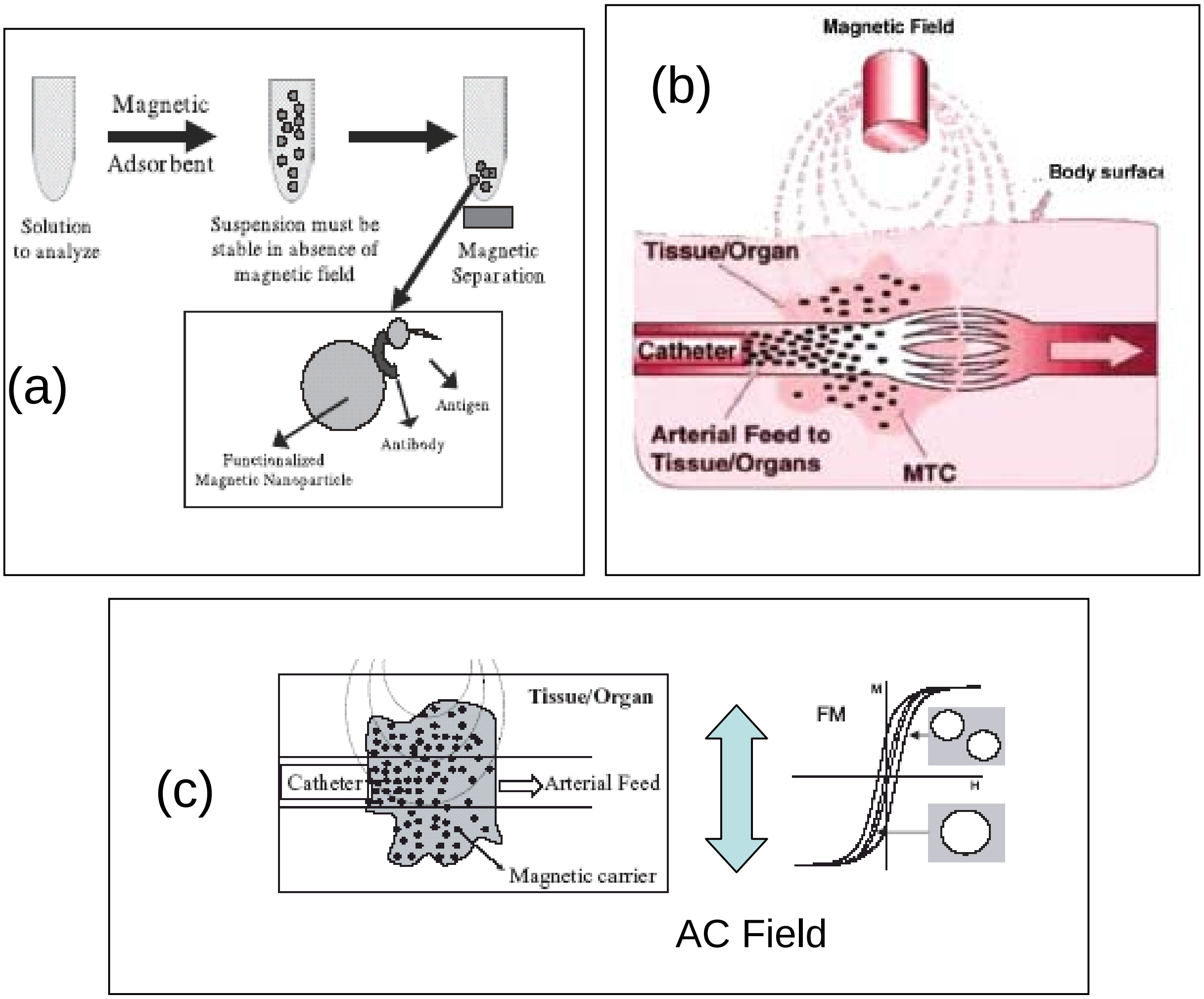

**FIGURE 20.** Scheme of the (a) cell separation, (b) drug delivery, and (c) hyperthermia using magnetic NP. (From refs. [5,132,134]). Reproduced with permission of the authors.

These NPs (or *nanoentities* formed by the NP, coating, and functional molecules) can be quite large. Therefore, when they move inside the biological tissue, the magnetic force must overcome the fluid viscosity that is proportional to the particle radius[132]. The magnetic force also increases with the volume (i.e., with $R^3$), but only with the volume of the magnetic core. Recent studies showed that the NP sizes must be limited in order to increase their effectiveness in biomedical applications. Large particles do not diffuse easily through intercellular spaces and tend to remain attached to the walls of the vascular system when put into the blood vessels. Thus, much effort is required in this field in order to increase the use of NPs in those biomedical applications. However, the potential advantages of using these NPs are so huge that all efforts put forth in this direction are worthy.

### CONCLUSIONS

In summary, it has been shown that decreasing particle size to the nanoscale leads to the appearance of new phenomena that are not observed at bulk scale, opening a wide world of applications of nanoparticles into different physical and chemical areas. The combination of new preparation techniques that allow us to obtain nanoparticles with narrow and well-defined size distribution profiles, with powerful characterization techniques, is giving a deep insight into the microscopic mechanisms responsible for that new phenomenology. In this way, it has been reported how the combination of size and surface effects could deeply modify the electronic structure of the nanoentities promoting a magnetic behavior completely unexpected in bulk state. The possibility of fabrication of tailored nanoparticles for a specific purpose, such as biomedical applications, opens a new era for material science.

### BIOSKETCHES

**Antonio Hernando** is Professor at the University Complutense of Madrid. He received his PhD at this same University in 1974. He founded and has been the Director of the Institute of Applied Magnetism at Madrid since 1989. He has been working on several fields of magnetism, both basic and research, as amorphous and nanocrystalline materials, magnetostriction, surface magnetism, magnetic semiconductors, and magnetic sensors. Recently, his research interest is focused on the magnetism at the nanoscale and the studies about the modification of magnetic properties of materials due to size effects. Other areas of interest are the effects of electromagnetic fields on health and the use of magnetic materials and electromagnetic fields in biomedical applications. In 1993, he was Invited Professor at Cambridge University, and he is Doctor Honoris Causa by the Bask Country University. He is a member of the Royal Spanish Academy of Science and among different awards, this year he received the Gold Medal of the Royal Spanish Physical Society.

**Patricia Crespo** achieved her PhD in Physics at the Complutense University of Madrid (1993). She is Assistant Professor at the Complutense University since 1996 and member of the research group of the Institude of Applied Magnetism at Madrid since 1989. Dr. Crespo worked as an invited researcher for 15 months at the Institut für Metallische Werkstoffe (I.M.W.) (I.F.W-Dresden), Germany. She was a member of the research team awarded with II Talgo prize for Technology Innovation (2001). Her research activities are focused on amorphous and nanostructured magnetic materials (solid solutions of immiscible elements and granular solids) and magnetic nanoparticles. In particular, she is working on the modification of magnetic properties of bulk materials by decreasing to the nanoscale and the biomedical application of such systems.

**Miguel Angel Garcia** is researcher at the Institute of Applied Magnetism. He received his PhD in Materials Science at the University Complutense of Madrid in 1999. The subject of his thesis was the study of optical properties of glasses containing metallic nanoparticles. From 2000 to 2001, he worked at the National Institute for Material Physics in Padua, Italy on semiconductor nanoparticles. Since 2002, he has worked at the Institute of Applied Magnetism at Madrid. His research interests are, in general, the modification of physical properties at the nanoscale and, in particular, the magnetic properties of nanostructures and magnetic semiconductors.
